# Prevalence and influencing factors of overweight and obesity in a Chinese rural population: the Henan Rural Cohort Study

**DOI:** 10.1038/s41598-018-31336-2

**Published:** 2018-08-30

**Authors:** Xiaotian Liu, Weidong Wu, Zhenxing Mao, Wenqian Huo, Runqi Tu, Xinling Qian, Xia Zhang, Zhongyan Tian, Haiqing Zhang, Jingjing Jiang, Yuqian Li, Chongjian Wang

**Affiliations:** 1 Department of Epidemiology and Biostatistics, College of Public Health, Zhengzhou University, Zhengzhou, Henan PR China; 2 Department of Occupational and Environmental Health Sciences, College of Public Health, Xinxiang Medical University, Xinxiang, Henan PR China; 3 Department of Occupational and Environmental Health Sciences, College of Public Health, Zhengzhou University, Zhengzhou, Henan PR China; 4 Department of Clinical Pharmacology, School of Pharmaceutical Science, Zhengzhou University, Zhengzhou, Henan PR China

## Abstract

The study aimed to estimate prevalence and influencing factors of overweight, general obesity, and abdominal obesity in rural areas of China. A total of 39034 participants aged 18 to 79 years were enrolled from the Henan Rural Cohort Study for the cross-sectional study. The age-standardized prevalence of overweight, general obesity, and abdominal obesity were 34.97%, 16.82%, and 43.71% in the general Chinese rural adults, respectively. Gender differences were: 36.04%, 18.98%, 35.37% for men, and 34.55%, 15.42%, 49.13% for women, respectively. The subgroup analysis showed the rates of overweight, general obesity, and abdominal obesity existed considerable disparities, but were universally high in all subgroups. Further, the study found that there were statistically significant U-shaped associations between the prevalence of overweight, general obesity, and abdominal obesity and age groups. In addition, the prevalence of participants with both abnormal BMI and WC were even at approximate forty percent. Aging, married/cohabiting, higher per capita monthly income, and unhealthy lifestyle were independent influencing factors of overweight, general obesity and abdominal obesity. In conclusion, overweight and obesity were severe in rural China. There is an increased need for closely monitoring high risk factors and promoting healthy lifestyle to curb the obesity epidemic among rural population.

## Introduction

As a disorder of energy metabolism, obesity affects multiple organ systems and is associated with a variety of vascular and several nonvascular complications, such as cardiovascular diseases (CVDs, mainly heart disease and stroke), diabetes, hypertension, dyslipidemia, musculoskeletal disorders, and some cancers^1–5^. Although the great efforts have been made to clear grim obesity in China, the prevalence of obesity still continues to increase, especially in rural areas with limited resources^6–9^. Moreover, the incidence rates are increasing faster in rural areas than those in cities^6,7^. The summary prevalence of general obesity and overweight in rural China were reported to be 3.6% and 8.2% in 1993. Whereas, the latest published nationwide prevalence estimates from 2010 have climbed to 11.0% and 29.1% for general obesity and overweight, respectively^8,9^. In addition, according to the 2011 China Health and Nutrition Survey (CHNS) data, the prevalence of abdominal obesity was 44.0% in rural China^10^. Therefore, ongoing reliable estimations are needed to plan effective national prevention and control programs for obesity management and reducing the disease complications in areas with limited resources.

Different anthropometric measures such as body mass index (BMI), waist circumference (WC), waist-to-hip ratio (WHR), and waist-to-height ratio (WHtR) have been proposed to define obesity. However, BMI is related to body fat percentage and could well reflect the degree of obesity deducting the influence of different heights while WC is the most practical and simplest index to measure the magnitude of the accumulation of fat in the abdomen. Thus, BMI and WC are the commonly-used anthropometric measurements for obesity in China^11–14^.

The aims of the current study were to provide the recent estimates of the prevalence of overweight, general obesity, and abdominal obesity, and to investigate potential influencing factors based on a large epidemiological survey conducted in Chinese rural adults, which will help policy making for obesity management in Chinese rural population.

## Results

### Demographic characteristics

Table 1 summaries the demographic characteristics of the 39034 participants aged 18–79 years old. The mean (standard deviation, SD) age was 55.61 (12.16) years. Overall, a total of 15446 and 6932 subjects were diagnosed with overweight and general obesity, respectively. The mean BMI were 25.85 kg/m^2^ for participants with overweight and 30.30 kg/m^2^ for obesity (*P* < 0.001). Current cigarette smoking, adequate vegetable and fruit intake, and lower level of height were more common among leaner study participants, while younger age, being women, married/cohabiting, lower education level, higher per capita monthly income, current alcohol drinking, high fat diet, lack of physical activity, higher level of weight, WC, and BMI were more prevalent among overweight and general obese participants.

**Table 1:** Demographic characteristics of the participants.

Variable	Overall (N = 39034)	BMI (N = 39034)				*χ*2*/F*	*P*	WC (N = 39034)			
		General obesity (n = 6932)	Overweight (n = 15446)	Normal weight (n = 15705)	Underweight (n = 951)			Abdominal obesity (n = 20167)	Normal (n = 18867)	*χ*2*/t*	*P*
Age (years), mean ± SD	55.61 ± 12.16	54.74 ± 11.42	55.62 ± 11.32	55.85 ± 12.94	57.73 ± 16.16	23.657	<0.001	56.05 ± 11.19	55.14 ± 13.10	7.403	<0.001
Sex, n (%)						117.717	<0.001			2796.344	<0.001
Women, n (%)	23615 (60.50)	4477 (64.58)	9541 (61.77)	9064 (57.71)	533 (56.05)			14753 (73.15)	8862 (46.97)		
Men, n(%)	15419 (39.50)	2455 (35.42)	5905 (38.23)	6641 (42.29)	418 (43.95)			5414 (26.85)	10005 (53.03)		
Marital status, n (%)						60.609	<0.001			0.302	0.583
Married/cohabiting	25048 (91.69)	6355 (92.84)	14074 (92.32)	13861 (90.80)	758 (87.03)			18311 (91.61)	16737 (91.77)		
Widowed/single/divorced/separation	3177 (8.31)	490 (7.16)	1170 (7.68)	1404 (9.20)	113 (12.97)			1676 (8.39)	1501 (8.23)		
Education, n (%)						20.361	<0.001			144.241	<0.001
≤Primary school	17469 (44.75)	3184 (45.93)	6784 (43.92)	7022 (44.71)	479 (50.37)			9615 (47.68)	7854 (41.63)		
≥Junior school	21565 (55.25)	3748 (54.09)	8662 (56.08)	8683 (55.29)	472 (49.63)			10552 (52.32)	11013 (58.37)		
Per capita monthly income, n (%)						86.542	<0.001			29.107	<0.001
≤500 RMB	13920 (35.66)	2281 (32.91)	5322 (34.46)	5905 (37.60)	412 (43.32)			6949 (34.46)	6971 (36.95)		
500 RMB ~	12837 (32.89)	2384 (34.39)	5229 (33.85)	4939 (31.45)	285 (29.97)			6822 (33.83)	6015 (31.88)		
≥1000 RMB	12277 (31.45)	2267 (32.72)	4895 (31.69)	4846 (30.95)	254 (26.71)			6396 (31.72)	5881 (31.17)		
Smoking, n (%)						216.362	<0.001			1647.513	<0.001
Never	28401 (72.76)	5315 (76.67)	11479 (74.32)	10945 (69.69)	662 (69.61)			16435 (81.49)	11966 (63.42)		
Ever	3175 (8.13)	567 (8.18)	1283 (8.31)	1244 (7.92)	81 (8.52)			1266 (6.28)	1909 (10.12)		
Current	7458 (19.11)	1050 (15.15)	2684 (17.38)	3516 (22.39)	208 (21.87)			2466 (12.23)	4992 (26.46)		
Drinking, n (%)						58.449	<0.001			546.729	<0.001
Never	30159 (77.26)	5334 (76.95)	11869 (76.84)	12170 (77.49)	786 (82.65)			16521 (81.92)	13638 (72.28)		
Ever	1820 (4.66)	259 (3.74)	702 (4.54)	808 (5.14)	51 (5.36)			641 (3.18)	1179 (6.25)		
Current	7055 (18.07)	1339 (19.32)	2875 (18.61)	2727 (17.36)	114 (11.99)			3005 (14.90)	4050 (21.47)		
Adequate vegetable and fruit intake, n (%)	16307 (41.78)	2710 (39.09)	6444 (41.72)	6773 (43.13)	380 (39.96)	33.630	<0.001	8081 (40.07)	8226 (43.60)	49.929	<0.001
High fat diet, n (%)	7449 (19.08)	1384 (19.97)	3009 (19.48)	2927 (18.64)	129 (13.56)	25.852	<0.001	3786 (18.77)	3663 (19.41)	2.599	0.107
Physical activity, n (%)						84.917	<0.001			159.083	<0.001
Low	12584 (32.24)	2504 (36.12)	4928 (31.90)	4814 (30.65)	338 (35.54)			6825 (33.84)	5759 (30.52)		
Moderate	14743 (37.77)	2519 (36.34)	5870 (38.00)	5977 (38.06)	377 (39.64)			7860 (38.97)	6883 (36.48)		
High	11707 (29.99)	1909 (27.54)	4648 (30.09)	4914 (31.29)	236 (24.82)			5482 (27.18)	6225 (32.99)		
Height (cm), mean ± SD	159.72 ± 8.20	159.46 ± 8.43	159.67 ± 8.18	159.88 ± 8.10	159.71 ± 8.59	4.560	0.003	159.04 ± 8.19	160.45 ± 8.15	−17.123	<0.001
Weight (kg), mean ± SD	63.50 ± 11.14	77.22 ± 9.65	66.07 ± 7.42	56.04 ± 6.71	44.84 ± 5.52	15227.926	<0.001	68.80 ± 10.60	57.83 ± 8.63	111.768	<0.001
BMI(kg/m^2^), mean ± SD	24.83 ± 3.57	30.30 ± 2.16	25.85 ± 1.12	21.87 ± 1.45	17.53 ± 1.00	62611.671	<0.001	27.12 ± 2.93	22.39 ± 2.37	174.127	<0.001
WC (cm), mean ± SD	84.08 ± 10.40	97.10 ± 7.52	87.07 ± 6.13	76.52 ± 6.58	65.47 ± 5.33	19677.480	<0.001	91.27 ± 7.56	76.40 ± 6.96	201.722	<0.001

BMI, body mass index; WC, waist circumference.

Among the 39034 participants, 20167 were diagnosed with abdominal obesity. The mean WC were 91.27 cm for participants with abdominal obesity and 76.40 cm for participants without abdominal obesity (*P* < 0.001). Compared with the subjects without abdominal obesity, the subjects with abdominal obesity had the following characteristics: older age, being women, higher percentage of lower education level, higher per capita monthly income, never cigarette smoking, never alcohol drinking, inadequate vegetable and fruit intake, high fat diet, physical inactivity, lower level of height, higher level of weight and BMI (*P* < 0.05 for each).

### The sex-specific distributions of BMI and WC according to age

**Figure 1:**
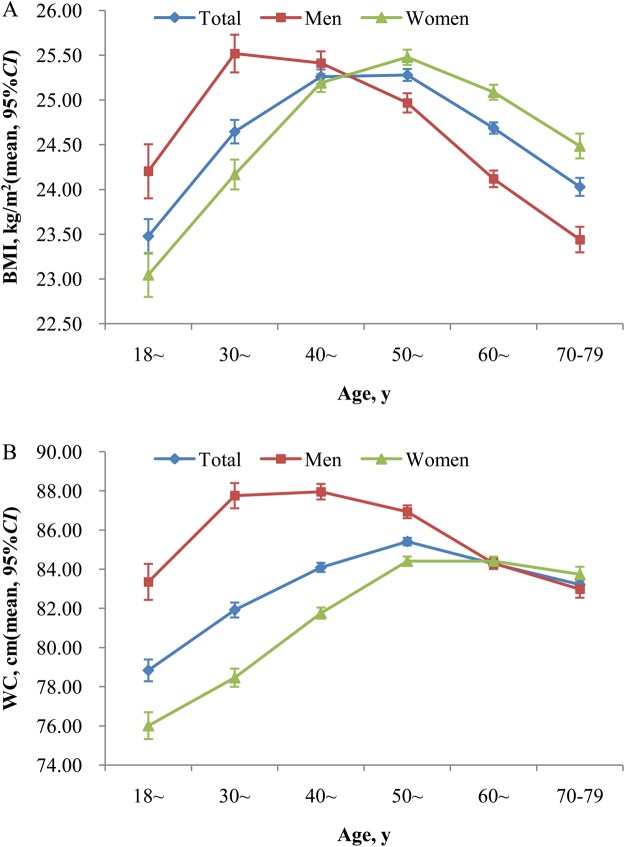
Mean (95%CI) of BMI and WC according to age in both sexes. Error bars indicate 95% confidence intervals. The mean was adjusted for age and sex in total population. All P values for U-shaped (quadratic term) association with age for BMI and WC in both sexes from the Curve Estimation <0.05. CI, confidence intervals; BMI, body mass index; WC, waist circumference.

Figure 1 displays the sex-specific distributions of BMI and WC according to age. The age- and sex-adjusted mean levels (95% confidence intervals, *CI*) of BMI and WC were 24.79 (24.75–24.82) kg/m^2^ and 84.34 (84.23–84.44) cm, respectively. The age-adjusted mean levels (95%*CI*) of BMI and WC were 24.56 (24.51–24.62) kg/m^2^ *vs*. 25.01 (24.97–25.06) kg/m^2^ and 85.55 (85.39–85.71) cm *vs*. 83.12 (82.99–83.25) cm for men and women (*P* < 0.001), respectively. There were statistically significant U-shaped associations between the mean levels of BMI, WC and age groups (All *P* < 0.05), with the highest mean values among study participants with age of 30 to 39 in men and 50 to 59 in women. In general, women had higher sex specific mean levels of BMI and WC than men at age above 50 years and 60 years, respectively.

### Prevalence of overweight and obesity

Table 2 describes the prevalence of overweight and obesity among various characteristics. The prevalence of overweight, general obesity, and abdominal obesity were 39.57%, 17.76%, and 51.67%, and the corresponding age-standardized rates were 34.97%, 16.82%, and 43.71%, respectively. Subgroups study showed that the prevalence of overweight, general obesity, and abdominal obesity were higher in those who were older, being female, married/cohabiting, with higher per capita monthly income, never cigarette smoking, current alcohol drinking, inadequate vegetable and fruit intake, high fat diet, and lack of physical activity, and were universally high in all subgroups (>26.00%, 13.00%, and 30.00%).

**Table 2:** Prevalence of overweight and obesity among various characteristics (N = 39034).

Variable	BMI (N = 39034)				WC (N = 39034)		
	General obesity (n = 6932)	Overweight (n = 15446)	*χ* ^2^	*P*	Abdominal obesity (n = 20167)	*χ* ^2^	*P*
Age, n (%)			903.493	<0.001		557.220	<0.001
18~	182 (14.03)	340 (26.21)			400 (30.84)		
30~	500 (18.08)	956 (34.56)			1157 (41.83)		
40~	1448 (19.89)	3098 (42.55)			3797 (52.16)		
50~	2154 (20.05)	4649 (43.28)			6247 (58.15)		
60~	2024 (16.56)	4780 (39.11)			6348 (51.94)		
70~79	624 (13.20)	1623 (34.33)			2218 (46.92)		
Sex, n (%)			117.717	<0.001		2796.344	<0.001
Women, n (%)	4477 (18.96)	9541 (40.40)			14753 (62.47)		
Men, n(%)	2455 (15.92)	5905 (38.30)			5414 (35.11)		
Marital status, n (%)			60.609	<0.001		0.302	0.583
Married/cohabiting	6355 (18.13)	14074 (40.16)			18311 (52.25)		
Widowed/single/divorced/separation	490 (15.42)	1170 (36.82)			1676 (52.75)		
Education, n (%)			20.361	<0.001		144.241	<0.001
≤Primary school	3184 (18.23)	6784 (38.83)			9615 (55.04)		
≥Junior school	3748 (17.38)	8662 (40.17)			10552 (48.93)		
Per capita monthly income, n (%)			86.542	<0.001		29.107	<0.001
≤500 RMB	2281 (16.39)	5322 (38.23)			6949 (49.92)		
500 RMB ~	2384 (18.57)	5229 (40.73)			6822 (53.14)		
≥1000 RMB	2267 (18.47)	4895 (39.87)			6396 (52.10)		
Smoking, n (%)			216.362	<0.001		1647.513	<0.001
Never	5315 (18.71)	11479 (40.42)			16435 (57.87)		
Ever	567 (17.86)	1283 (40.41)			1266 (39.87)		
Current	1050 (14.08)	2684 (35.99)			2466 (33.07)		
Drinking, n (%)			58.449	<0.001		546.729	<0.001
Never	5334 (17.69)	11869 (39.35)			16521 (54.78)		
Ever	259 (14.23)	702 (38.57)			641 (35.22)		
Current	1339 (18.98)	2875 (40.75)			3005 (42.59)		
Adequate vegetable and fruit intake, n (%)	2710 (16.62)	6444 (39.52)	33.630	<0.001	8081 (49.56)	49.929	<0.001
High fat diet, n (%)	1384 (18.58)	3009 (40.39)	25.852	<0.001	3786 (50.83)	2.599	0.107
Physical activity, n (%)			84.917	<0.001		159.083	<0.001
Low	2504 (19.90)	4928 (39.16)			6825 (54.24)		
Moderate	2519 (17.09)	5870 (39.82)			7860 (53.31)		
High	1909 (16.31)	4648 (39.70)			5482 (46.83)		

### Changes of overweight and obesity in different subgroups

**Figure 2:**
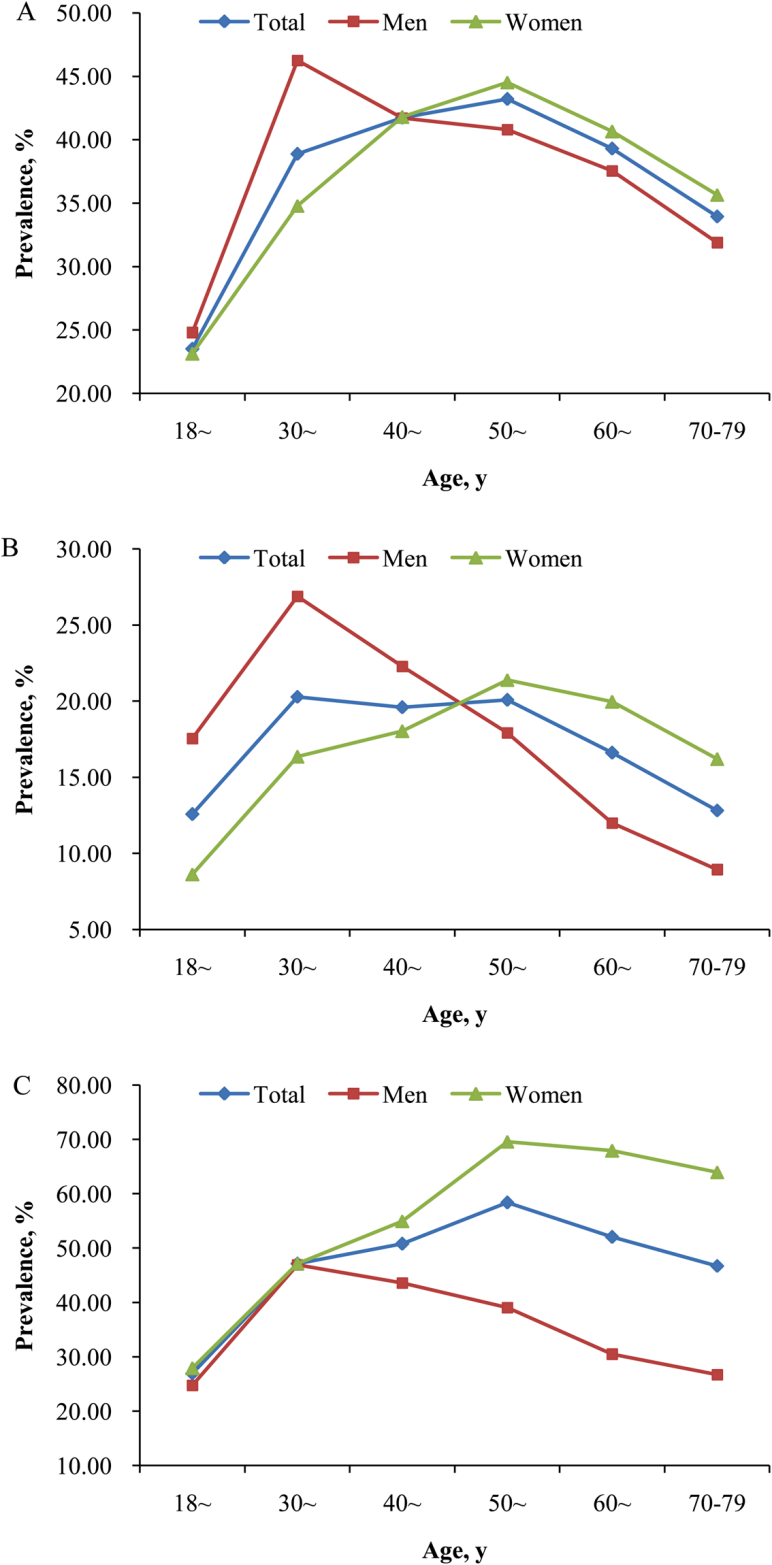
Changes in the age-standardized prevalence of overweight and obesity with aging in different sexes. **(A)** is for overweight, **(B)** is for general obesity, (**C**) is for abdominal obesity. All P values for U-shaped (quadratic term) association with age for overweight and obesity in both sexes from the Curve Estimation <0.05.

Figure 2 presents that the age-standardized prevalence of overweight and obesity displayed U-shaped association with aging in both sexes (All *P* < 0.05). The age-standardized prevalence of overweight, general obesity, and abdominal obesity in men and women were 36.04% vs. 34.55%, 18.98% vs. 15.42%, and 35.36% v*s*. 49.12%, respectively. The prevalence of overweight, general obesity, and abdominal obesity increased with age until 40 years in men and 60 years in women and then decreased. Women had a higher prevalence of overweight than men at age above 40 years, but lower prevalence when they were in midlife and youth (Fig. 2A). Figure 2B implies that men had more probability of having general obesity than women except the highest two age groups. As to abdominal obesity, women had higher prevalence than men in the entire age ranges except the age group of 30 to 39 (Fig. 2C).

### The percentage according to the cut-off points of BMI and WC in different sexes

**Figure 3:**
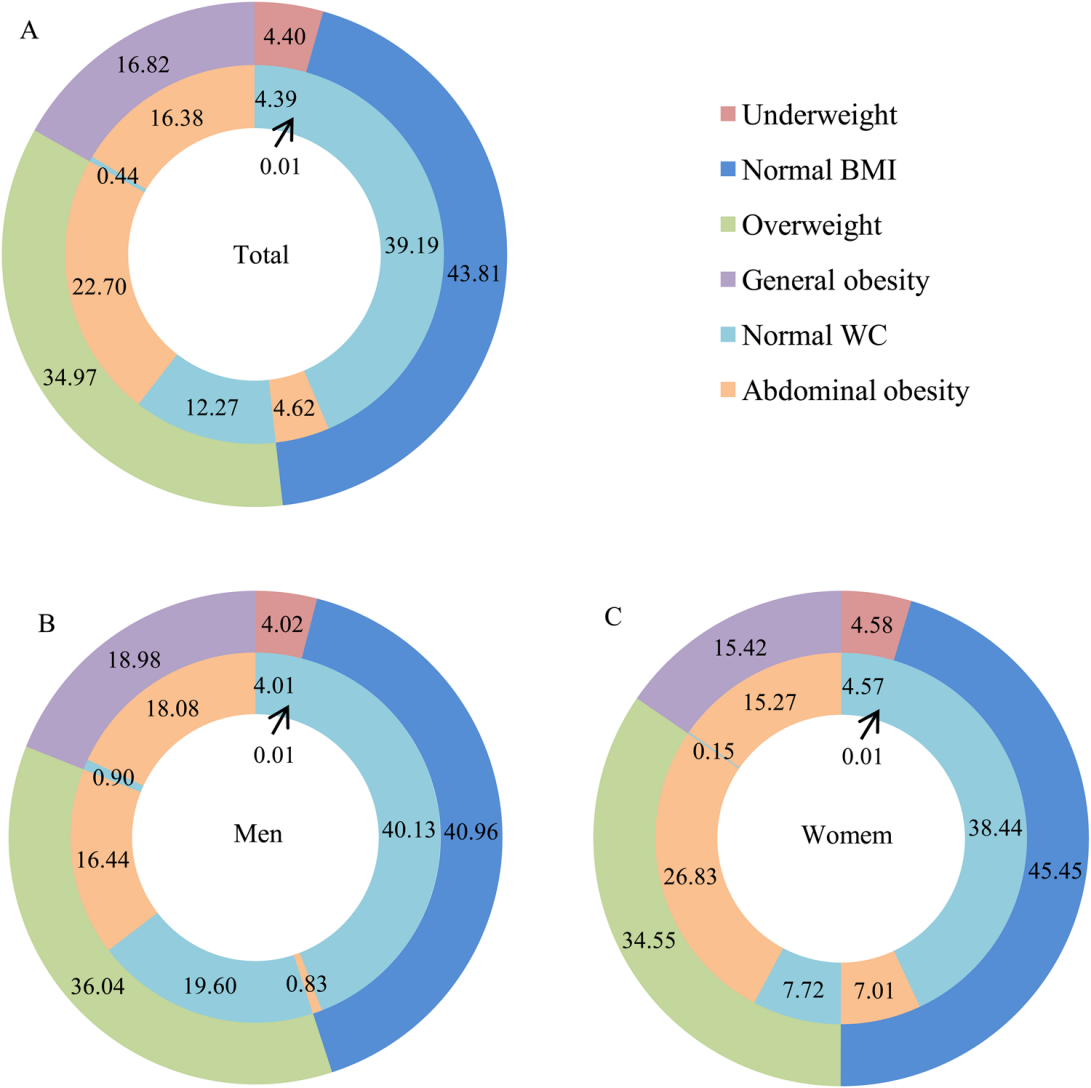
The age-standardized percentage according to the cut-off points of BMI and WC in different sexes. BMI, body mass index; WC, waist circumference.

Figure 3 shows the age-standardized percentage according to the cut-off points of BMI and WC in different sexes. A total of 24902 subjects (8567 men and 16335 women) had excess BMI or WC, and the corresponding age-standardized prevalence was 56.41% (55.85% in men and 56.98% in women). In detail, 4619, 123, 2524, 10827, and 6809 were diagnosed with single overweight, single general obesity, single abdominal obesity, abdominal obesity &overweight, and abdominal obesity & general obesity, and the corresponding age-standardized prevalence were 12.27%, 0.44%, 4.62%, 22.70%, and 16.38%, respectively. The age-standardized prevalence of participants with both excess BMI and abdominal obesity was 39.08% (34.52% for men and 42.10% for women). The prevalence of general obesity and abdominal obesity differed depending on the cut-offs of BMI or WC in both sexes. Among the participants with a normal BMI, 10.55% (2.02% for men and 15.42% for women) still had abdominal obesity. Likewise, among participants with general obesity, 2.62% (4.74% for men and 0.97% for women) had a normal WC.

### Analysis of influencing factors

Table 3 describes the odds ratios (*ORs*) of potential influencing factors associated with overweight, general obesity and abdominal obesity. Old age, married/cohabiting, higher per capita monthly income, never cigarette smoking, current alcohol drinking, inadequate vegetable and fruit intake, high fat diet, and lack of physical activity were significantly positively associated with the prevalence of overweight, general obesity and abdominal obesity. Men were negatively associated with the prevalence of general obesity and abdominal obesity. Higher education level was only related to general obesity.

**Table 3:** Associations of potential risk factors for overweight and obesity.

Variable	Overweight	General obesity	Abdominal obesity
	*OR(95%CI)*	*OR(95%CI)*	*OR(95%CI)*
Age			
18~	1.00	1.00	1.00
30~	1.39 (1.17–1.65)	1.45 (1.17–1.79)	1.52 (1.30–1.77)
40~	2.13 (1.82–2.49)	1.93 (1.58–2.35)	2.42 (2.09–2.80)
50~	2.29 (1.97–2.67)	2.09 (1.72–2.54)	3.27 (2.83–3.77)
60~	1.79 (1.53–2.10)	1.41 (1.15–1.72)	2.73 (2.36–3.15)
70~79	1.40 (1.18–1.65)	0.96 (0.78–1.19)	2.26 (1.94–2.64)
*P*_trend_	0.748	<0.001	<0.001
Gender			
Women	1.00	1.00	1.00
Men	0.94 (0.87–1.02)	0.87 (0.79–0.96)	0.30 (0.28–0.32)
Marital status			
Married/cohabiting	1.00	1.00	1.00
Widowed/single/divorced/separation	0.89 (0.82–0.97)	0.86 (0.77–0.96)	0.90 (0.83–0.97)
Education			
≤Primary school	1.00	1.00	1.00
≥Junior school	0.98 (0.93–1.03)	0.82 (0.77–0.88)	0.96 (0.91–1.01)
Per capita monthly income			
≤500 RMB	1.00	1.00	1.00
500 RMB~	1.13 (1.07–1.19)	1.17 (1.09–1.25)	1.12 (1.07–1.18)
≥1000 RMB	1.08 (1.02–1.14)	1.14 (1.05–1.22)	1.13 (1.07–1.19)
*P*_trend_	0.008	0.001	<0.001
Smoking			
Never	1.00	1.00	1.00
Ever	0.94 (0.84–1.04)	0.95 (0.83–1.09)	1.03 (0.94–1.14)
Current	0.67 (0.61–0.72)	0.56 (0.50–0.63)	0.77 (0.71–0.84)
Drinking			
Never	1.00	1.00	1.00
Ever	1.09 (0.97–1.23)	1.03 (0.87–1.21)	1.08 (0.96–1.21)
Current	1.33 (1.24–1.43)	1.56 (1.42–1.72)	1.53 (1.43–1.64)
Adequate vegetable and fruit intake	0.93 (0.88–0.97)	0.84 (0.79–0.89)	0.87 (0.84–0.91)
High fat diet	1.06 (1.00–1.13)	1.12 (1.04–1.20)	1.19 (1.13–1.26)
Physical activity			
low	1.00	1.00	1.00
Moderate	0.89 (0.84–0.94)	0.73 (0.68–0.78)	0.79 (0.75–0.83)
High	0.86 (0.81–0.91)	0.68 (0.64–0.74)	0.71 (0.67–0.75)
*P*_trend_	<0.001	<0.001	<0.001

*OR:* odds ratios, *CI:* confidence intervals.

## Discussion

The present large survey specialized in Chinese rural population provided important new evidence on the current burden of overweight and obesity in China. Overall, the mean levels of BMI, WC, and prevalence of overweight, general obesity, and abdominal obesity were much higher in Chinese rural adults than the previous national studies in China^6–8^. Men had more likely to be placed in the categories of general obesity, while women were more probability of being abdominal obesity. There were statistically significant U-shaped associations between the mean levels of BMI and WC and age groups, with the highest mean values among study participants with age of 30 to 39 in men and 50 to 59 in women. Similar significant trends were observed in the prevalence of overweight, general obesity, and abdominal obesity in both men and women. Subgroup analysis found that the proportions of overweight, general obesity, and abdominal obesity varied significantly across subpopulations, and were universally high in all subgroups (>26.00%, 13.00%, and 30.00%). Further study indicated that approximately forty percent of the rural adults in China had both excess BMI and WC. The multiple logistic regression analysis showed that old age, married/cohabiting, higher per capita monthly income, never cigarette smoking, current alcohol drinking, inadequate vegetable and fruit intake, high fat diet, and lack of physical activity were associated influencing factors for overweight, general obesity and abdominal obesity.

Evidences from previous epidemiological studies showed that BMI and WC levels had increased dramatically in Chinese populations^6,7,13,15^. Similar results were found in our study. The mean levels (95%*CI*) of BMI and WC were 24.79 (24.75–24.82) kg/m^2^ and 84.34 (84.23–84.44) cm, respectively. Despite of adjusting for sex and age, the mean levels of BMI and WC documented a 7.32% (or 1.69 kg/m^2^) and 5.95% (or 4.74 cm) increase compared with the national data in the study of Reynolds *et al*.^6^ conducted in 2000–2001, respectively. Due to changes in BMI and WC levels, the prevalence of overweight, general obesity, and abdominal obesity have been increasing in the past decades, and overweight and obesity have become the most important public health problems due to their common associations with many chronic diseases in China^2,4,5,8,15^. Although China brought obesity and overweight into the non-communicable disease (NCD) targets, the rates of overweight, general obesity, and abdominal obesity still increased rapidly in the Chinese population, especially in rural population^7,8,15^. The overall age-standardized prevalence of overweight and general obesity in current study were obviously higher than those in rural areas of the China in 2010 although there was no difference in prevalence of abdominal obesity between the current study and the CHNS in 2011^9,10^. However, the subgroup analysis showed that the rates of overweight, general obesity, and abdominal obesity were universally high in all subgroups. Thus, effective measures should be taken to improve the status of the prevention and control of obesity and overweight, especially in rural areas with limited health care resources.

Data from Working Group on Obesity in China (WGOC) shows that the subjects with excess BMI or WC, especially with the aggregation of excess BMI and WC, were more susceptible to diabetes, hypertension, dyslipidemia, and the accumulation of risk factors compared to people with normal BMI and WC^11^. In the current study, exceeded fifty five percentages of the rural adults had excess BMI or WC in China, and the prevalence of participants with both abnormal BMI and WC were even approximately forty percent. Therefore, effective preventive measures are needed to maintain both BMI and WC levels close to normal and decrease the incidence of overweight and obesity as well as related complications. In addition, the prevalence of excess BMI and abdominal obesity differed depending on the cut-off points of BMI or WC in both sexes. There was a considerable number of participants with a normal BMI had abdominal obesity. Likewise, among participants with overweight and general obesity, there was still a considerable number who had normal WC. Therefore, it seems that it’s unreasonable to use only one indicator as the cardiovascular risk factor. The combination use of BMI and WC might be the best marker for indirectly reflecting cardiometabolic risk^11,12^.

Previous studies have declared that the prevalence of overweight and general obesity were higher in men than women while abdominal obesity was more prevalent in women than men^8,15^. Similar results were found in our study. The age-standardized prevalence of overweight, general obesity, and abdominal obesity in men and women were 36.04% vs. 34.55%, 18.98% vs. 15.42%, and 35.36% v*s*. 49.12%, respectively. Further study showed that there were different change trends in the prevalence of overweight, general obesity, and abdominal obesity in men and women. Women had obviously higher prevalence of overweight and general obesity than men at age above 50 years, but lower prevalence when they were in midlife and young. As to abdominal obesity, women had higher prevalence than men in the entire age ranges except the age group of 30 to 39. The phenomenon might attribute to the different cut-off values of WC in men and women. But the prevalence of overweight, general obesity, and abdominal obesity were obviously higher in women than men above 50 years of age. Age and the rapid hormone changes during the menopausal transition might explain for the BMI and WC distribution changes in women^6,13,16^. Moreover, Men aged 30 to 39 years had the highest prevalence, whereas women aged 50 to 69 years had the highest prevalence. These data all implied that the overweight and obesity management guidelines in our country should post middle-aged men and postmenopausal women as the priority management groups. Previous epidemiological studies have found that unhealthy lifestyle, such as alcohol drinking, inadequate vegetable and fruit intake, high fat diet, and lack of physical activity, was associated with the prevalence of overweight and obesity^3,11^. Our results were in accordance with the findings. With rapid economic growth, Chinese food consumption patterns have changed from predominantly rice, wheat and related products to high animal food consumption, frequency of alcohol consumption increased, and physical activities were reduced due to advances in technology and transportation^3,8,11,17,18^. Those dietary pattern and lifestyle changes might account for the major proportion of the increase in overweight, general obesity and abdominal obesity among Chinese adults^3,8,11,18^. Therefore, we should use mass media and government programs to encourage retention of its high-dietary fiber, low-fat, low-energy density traditional cuisine, moderate alcohol drinking, and sufficient physical activity life^3,11^. Furthermore, the emphasis of healthy education should be different for different high-risk populations. For men, a good lifestyle (e.g. no cigarette smoking or alcohol drinking), healthy diet (e.g. adequate vegetable and fruit intake and low fat diet), as well as exercise are also crucial in controlling obesity. For pregnant and breastfeeding women, promotion of breast feeding and healthy complementary feeding according to WHO recommendations is the most importance. For perimenopausal women, the key points of primary health care should be put on diet diversity and physical activity, such as increase consumption of fruit and vegetables, as well as legumes, whole grains and nuts; engage in regular physical activity; and keep good mood. As for wealthier populations, low-energy diets are effective in the short term, but reducing inactivity, increasing walking, and developing an activity programme might increase the effectiveness of obesity therapy. As for people with unhealthy lifestyle, lifestyle changes were effective in controlling body weight and WC^3,11^. The relationship between smoking status and body shape remains controversial. The current finding that the current cigarette smokers were generally leaner than never smokers was in agreement with most previous studies^19^. In contrast, there was no or positive association between smoking and body weight in some studies^20,21^. The reasons for the inconsistent association between smoking and higher BMI remained unclear. Unhealthy lifestyle (such as increased frequency of alcohol consumption, physical inactivity, and so on) or the different social classes might be partially responsible for the inconsistency^16^.

The current study synthesized the epidemiologic characteristics and influencing factors of overweight and obesity based on a relatively large sample size of rural population in China. The standardized survey tools, training and field implementation, and adjusting for a wide range of potential confounders guarantee the reliability of the analysis. However, several limitations should also be addressed. Firstly, these findings were derived from a cross-sectional study not a prospective cohort design, no causal relationships could be precisely delineated. Secondly, some residents such as college students and migrant workers were not included in the current study for studying or working outside. These groups were more likely to be young and healthy, with lower prevalence of overweight and obesity, which might lead to the overestimation of overweight and obesity in the rural population. Finally, the results were based on one geographical region in the middle area of China, which might not be a representative sample of the Chinese rural population. However, the rural population of Henan province accounts for 9% of rural Chinese population. Therefore, the results from the relatively large rural epidemiological study, to some extent, could reflect the prevalence of overweight and obesity in Chinese rural areas.

In conclusion, overweight and obesity have become one of the severe public problem in rural China. Therefore, closely monitoring high risk factors and promoting healthy lifestyle are essential to curb the obesity epidemic in the population including older, middle-aged men, postmenopausal women, people with unhealthy lifestyle in Chinese rural areas.

## Methods

### Study subjects

The Henan Rural Cohort Study was conducted in Yuzhou county, Suiping county, Xinxiang county, Kaifeng county, and Yima county of Henan province in China from July 2015 to September 2017, which has been registered in Chinese Clinical Trial Register (Registration number: ChiCTR-OOC-15006699) before the onset of patient enrollment^22^. The target population was permanent residents aged 18–79 years who lived in the five rural areas. A multistage, stratified cluster sampling method was used to select the sample. In the first stage, 5 counties were selected from different geographical regions (central, south, north, east, and west) in Henan Province in consideration of the adherence of the masses and local medical conditions. In the second stage, 1~3 typical rural communities (referred to as a “township”) in each county were selected by the local Centre for Disease Control and Prevention. In the final stage, all permanent residents who satisfied the inclusion criteria and signed informed consent in each sampled rural district were selected as the study sample. Overall, a total of 39259 participants aged 18–79 years old in rural areas completed the survey, with a response rate of 93.7%. To estimate the prevalence of overweight, general obesity, and abdominal obesity overall and by age, sex, and other demographic characteristics, and to explore potential influencing factors in rural areas, 144 who lacked weight, height, and WC data, 30 who were on a diet or took diet teas or diet pills, and 51 who were pregnant were excluded. Finally, 39034 subjects were included for the present study. Written informed consent was obtained from each participant before data collection. The Henan Rural Cohort Study was approved by the Zhengzhou University Life Science Ethics Committee (Code: [2015] MEC (S128)), and was conducted according to the 1975 Declaration of Helsinki.

### Assessment of covariates

The detailed information on demographic characteristics, smoking, alcohol drinking, dietary habits, physical activities, personal history of disease, and family history were collected through face-to-face interview by the trained research staff using a standardized questionnaire. Education level was classified into two categories: participants attained up to primary school level were considered as ‘≤Primary school’, while those attained junior school or higher levels of education were defined as ‘ ≥Junior school’. Smoking status was classified into current smoker (a person who smoked more than one cigarette per day in the past 6 months), ever smoker (a person who ever smoked) and never smoker. Alcohol drinking status was categorized into current drinking (a person who consumed twelve or more alcoholic drinks in the past one year, whether spirits, beer, wine or other forms of alcohol beverage), ever drinking (a person who ever drank), and never drinking. According to Chinese dietary guidelines^15^, adequate vegetable and fruit intake was considered as a person who consumed an average of more than 500 g vegetable and fruit per day. High fat diet was defined as a person who took an average of more than 75 g meat of livestock and poultry per day in accordance with the dietary guidelines for Chinese residents^23^. According to the international physical activity questionnaire (IPAQ 2001), physical activity was categorized into low, moderate, and high^24^.

### Assessment of outcomes

Body weight with light clothing was measured to the nearest 0.1 kg using a weight measurement device (V. BODY HBF-371, OMRON, Japan). Height was measured to the nearest 0.1 cm without shoes using a standard right-angle device and a fixed measurement tape. WC was measured at a point midway between the lowest rib and the iliac crest in a horizontal plane to the nearest 0.1 cm using non-elastic tape. All measurements were conducted twice by trained research staff according to a standard protocol from WGOC^11^, and the mean values were used for the statistical analysis. BMI was estimated as body weight (kg) divided by height square (m^2^). According to the criteria recommended by WGOC, underweight was defined as BMI < 18.5 kg/m^2^, normal weight was defined as 18.5 ≤ BMI < 24 kg/m^2^, overweight was defined as 24.0 kg/m^2^ ≤ BMI < 28.0 kg/m^2^, and general obesity was defined as BMI ≥ 28.0 kg/m^2^^11^. Abdominal obesity was defined according to guidelines of the International Diabetes Federation for Chinese populations as a WC ≥ 90 cm for men and ≥80 cm for women. Normal WC was defined as a WC <90 cm for men and <80 cm for women^25^.

### Statistical analysis

Continuous variables presented as mean ± SD were compared using the *t*-test or analysis of variance, while categorical variables presented as numbers and proportions were compared using chi-square test. The age- or sex-adjusted means (95%*CI*) of BMI and WC overall and in different sexes were estimated and compared by multiple linear regression. The stratified subgroup analyses for prevalence of overweight, general obesity, and abdominal obesity were performed according to the demographic characteristics. The presence of a U-shaped association between age group and overweight and obesity was tested using quadratic term of age group as a continuous variable in the curve estimation. The prevalence of overweight, general obesity, and abdominal obesity were standardized using the direct method according to the Chinese Population Census 2010^26^. The multivariable logistic regression models were used to calculate *OR* and 95%*CI* between the potential influencing factors and the prevalence of overweight, general obesity, and abdominal obesity. All selected characteristics were included in multivariable logistic regression models. Besides, to explore whether there had a linear trend of *ORs* between age groups, per capita monthly income, physical activity and obesity, the three indices were taken as continuous variables. All tests were two-tailed and a *P* value < 0.05 was the threshold for statistical significance. All statistical analyses were performed using SAS9.1 software package (SAS Institute, USA).

### What is already known on this subject?

Previous studies have concentrated on prevalence of obesity in China, but the studies focusing on epidemiology and risk factors in areas with limited resources are still limited. More importantly, little is known about the recent epidemiology in overweight, general obesity, and abdominal obesity in rural areas of China.

### What does this study add?

Prevalence and risk factors associated obesity assessed by two metrics were explored in the current study. The mean levels of body mass index (BMI) and waist circumference (WC) were high, and the prevalence of overweight, general obesity, and abdominal obesity were severe overall and in various subgroups in Chinese rural adults. Further, the study found that there were statistically significant U-shaped associations between the prevalence of overweight, general obesity, and abdominal obesity and age groups (All *P* < 0.05), with the highest prevalence among study participants with age of 30 to 39 in men and 50 to 59 in women, respectively. In addition, the prevalence of participants with both abnormal BMI and WC were approximately forty percent. Aging, married/cohabiting, higher per capita monthly income, and unhealthy lifestyles were independent influencing factors of overweight, general obesity and abdominal obesity. In conclusion, overweight and obesity were severe in rural China. Closely monitoring high risk factors and promoting healthy lifestyle are essential to curb the obesity epidemic among rural population.

The current study provides a contextual epidemiological analysis of overweight, general obesity, and abdominal obesity prevalence information for rural China, with a multiple regression analysis, to determine the strength of associations between obesity, overweight, and demographic and risk factors for the rural adults.
